# Age and Socioeconomic Gradients in Frailty Among Older Adults in India

**DOI:** 10.1093/geroni/igaa057.350

**Published:** 2020-12-16

**Authors:** Ravindra Chowdhary

**Affiliations:** Tata Institute of Social Sciences, Mumbai, Maharashtra, India

## Abstract

Although individuals with frailty and lower socioeconomic status (SES) are vulnerable to morbidity and early mortality, few studies have investigated this association. We intend to fill this gap with a study using older adults aged ≥ 50 years from the SAGE WAVE II in India. The Aim of study is to examine the association of frailty with SES and how this association varies across different age groups. A modified Fried phenotype approach with five frailty indicators was used to categorize 6560 older adults as frail, pre-frail or robust who had more than two, one or zero indicators, respectively: grip strength, exhaustion, weight loss, walking speed and physical activity. Multinomial logistic regression estimated the likelihood of being pre-frail and frail for various levels of SES, controlling and not controlling for confounders. This study also shows the overall socioeconomic gradients and age patterns of socioeconomic gradients of frailty indicators using predicted probabilities. Approximately 26%, 55% and 20% participants were robust, pre-frail and frail, respectively. The number of frailty indicators was positively associated with lower income and education levels in the case of controlling and not controlling for confounders. Also, among the higher age groups, individuals with low SES had higher chances of being frail.Overall, the results in this research indicated a negative low SES and frailty association as found in previous studies worldwide. This highlights the need for comprehensive and centered public health interventions for older adults with low SES.

